# Is the Processing of Chinese Verbal Metaphors Simulated or Abstracted? Evidence From an ERP Study

**DOI:** 10.3389/fpsyg.2022.877997

**Published:** 2022-07-13

**Authors:** Ying Li, Xiaoxiao Lu, Yizhen Wang, Hanlin Wang, Yue Wang

**Affiliations:** ^1^School of Education, Zhengzhou University, Zhengzhou, China; ^2^School of Psychology, Central China Normal University, Wuhan, China; ^3^School of International Studies, Zhengzhou University, Zhengzhou, China; ^4^Department of Psychology, Hebei Normal University, Shijiazhuang, China

**Keywords:** verbal metaphor, simulation, abstraction, N400, P600/LPC

## Abstract

The theory of embodied semantics holds that verbal metaphors are strongly grounded in sensorimotor experience. Many studies have proven that besides sensorimotor simulation, the comprehension of verbal metaphors also requires semantic abstraction. But the interaction between simulation and abstraction, as well as the time course of metaphorical meaning integration, is not well understood. In the present study, we aimed to investigate whether embodiment or abstraction, or both, is employed in the processing of Chinese verbal metaphor. Participants were asked to read subject-verb metaphorical, verb-object metaphorical, literal-concrete and literal-abstract sentences, and the target words were measured at the verb and the object of each sentence. The results revealed that a similar N400 effect was elicited by the target verbs in the verb-object metaphorical and the literal-concrete sentences, and a similar P600/LPC effect was induced by the target verbs in the subject-verb metaphorical and the literal-abstract sentences, reflecting that the verb-object metaphors trigger a simulation process, while the subject-verb metaphors trigger an abstraction process in the verb processing stage. Moreover, the subject-verb metaphors elicited a stronger P600/LPC effect by the target verbs than the verb-object metaphors, but there was no difference of the P600/LPC caused by the target objects between the two kinds of metaphors, revealing that the metaphorical meaning of a subject-verb metaphor is integrated in the verb processing stage, while that of a verb-object metaphor is reanalyzed in the object processing stage. These results suggest that a verbal metaphor is processed both by simulation and abstraction, and the metaphorical meaning is integrated immediately with the unfolding of the sentence meaning. The position where the semantic conflict lies in a sentence (verb vs. object) modulates the time course of metaphor sentence comprehension.

## Introduction

A metaphor refers to using the flexibility of semantic features to express some new meanings by forming semantic conflict with its literal meaning (Semino et al., 2008; Rutter et al., 2012; Benedek et al., 2014). Unlike literal languages, in metaphorical expressions, when a sentence produces such a semantic conflict, the novel meaning created is not inappropriate but conveys a figurative, abstract sense. Dedre Gentner and France (1988) suggested that verbal metaphors would be generated when the collocation of a verb and a noun is unconventional. For example, in the expression “The rumor flew through the office,” the subject of the action verb “fly” is an abstract and inanimate agent “rumor” which cannot physically perform the action of “fly.” Therefore, the agent (e.g., rumor) and the verb (e.g., fly) constitute a strong conflict, producing a metaphorical expression that means “rumor spreads very fast.” Similarly, it is clear that when someone says “he catapulted his words from the dais,” the speaker does not literally mean that the orator uses a catapult to bombard the audience, but rather that s/he speaks with vehemence (Obert et al., 2018). Verbal metaphor is not only frequently used in daily life and literary works (Cameron and Gibbs, 2008), but also leads to a wide range of scientific research on how action-based metaphors are comprehended.

A verbal metaphor is a linguistic construction that exemplifies the embodied nature of cognition as verbs generally entail more action content (Feng and Zhou, 2021). The embodied semantic view of metaphors holds that the processing of verbal metaphors relies on sensorimotor simulation, as the verbal metaphors are grounded in the physical body and sensorimotor system (Barsalou et al., 2003; Gibbs, 2006; Qu et al., 2013; Yin et al., 2013). From this perspective, to understand a verbal metaphor such as “The media bent the truth,” we need to simulate the concrete act of “causing to curve.” Wilson and Gibbs (2007) found that people are faster to assess whether or not a sentence is meaningful if they perform or imagine performing a congruent motion before reading a metaphor (e.g., a grasp motion before reading “grasp a concept”), suggesting that comprehension of the metaphorical term “grasp” draws from simulation of its literal meaning. The embodied simulation view has been supported recently by neural studies. It has been demonstrated that there are sensorimotor activations across brain regions when participants read verbal metaphors (Desai et al., 2011; Boulenger et al., 2012; Lauro et al., 2013; Lai et al., 2019). For example, reading verbal metaphors related to motion content (e.g., grasping a concept) can activate brain regions involved in motor perception and planning associated with hands (Desai et al., 2013; Lauro et al., 2013). A few secondary motor regions are found to be involved when participants read familiar verbal metaphors, while the primary sensory and motor regions are more active when they read unfamiliar novel metaphors (Desai et al., 2011, 2013; Cardillo et al., 2012), suggesting that novel predicate metaphors rely more on sensorimotor information corresponding to the verbs. To sum up, these studies support the view of embodied simulation that verbal metaphor comprehension counts on sensorimotor simulation, and reading a verbal metaphor activates the sensorimotor system associated with the embodied experiencing of concrete conceptual domains of the metaphor.

However, in the psycholinguistic literature on this topic, the dominant assumption is that metaphorical representation is processed as abstractions rather than concrete representations. Abstractions are conceptual representations that are less specified (Gentner and Asmuth, 2019) than their literal-concrete counterparts. According to this view, verbal metaphor comprehension is a result of abstraction processing of the semantic system, where literal-level concrete features of a concept play a little-to-no role in metaphor comprehension. Such as, when reading the familiar expression “The media bent the truth,” one would directly retrieve the abstract meaning “distort” from long-term memory. Compared with literal sentences that convey physical senses (e.g., The repairman bent the pipe), verbal metaphors use verbs figuratively *via* abstraction from concrete action terms. Desai et al. (2011) found that the activation of sensorimotor regions decreases with the increase of sentence abstraction. Raposo et al. (2009) reported more specifically that when verbs are presented in literal-concrete sentences, there is greater activation in the sensorimotor areas and frontotemporal lobes associated with language processing. However, when verbs are presented in metaphorical sentences, there is no activation in the motor and premotor areas. Meanwhile, Chatterjee (2010) also proved that understanding a literal sentence with an action verb activates the left occipital and temporal motor area. Instead, when reading a metaphorical sentence with the same verb, the inferior frontal gyrus and left temporal gyrus relating to language are more active, indicating that the semantic abstraction system is more involved when dealing with verbs in metaphors.

Although the activation of abstract meaning in metaphorical processing has been reported in a few studies, it is unclear how the abstract sense and the concrete one interact and work together to access the metaphorical meaning. If the comprehension of verbal metaphors is to combine the neural patterns of literal-concrete meaning processing and literal-abstract one, it is pressing to explore at what stage the concrete and abstract meanings in verbal metaphors are activated. In other words, are the verbal metaphors comprehended in their concrete sense first, or can metaphorical meaning be extracted directly through semantic abstraction? If literal-concrete meaning activation is early, then it would support the critical role of concrete and bodily experiences in comprehending abstract meaning (Gallese and Lakoff, 2005). In contrast, if it is activated late, then such activation can be interpreted as being epiphenomenal, suggesting that metaphorical meaning is accessed directly through semantic abstraction (Mahon and Caramazza, 2008). Therefore, the present study intended to use Event-Related Potentials (ERP) to investigate the activation timings of literal-concrete and literal-abstract meaning in metaphorical sentences, which can reveal whether the processing of verbal metaphors is simulated or abstracted.

Previous research adopted electrophysiological indexes to detect the processing of the verbal metaphors, and highlighted that the N400 may reflect activation of concrete word’s multimodal information, which is related to sensorimotor recruitment. For example, Lai et al. (2019) found that when the verb presented, both metaphorical and literal sentences including concrete action verbs elicited larger N400 effect than the sentences containing abstract verbs. Holcomb et al. (1999) suggested that more semantic information is activated in the long-term semantic memory *via* concrete words than abstract words. However, when the semantic association strengths were controlled constant for concrete and abstract words, the concreteness N400 effect was still found in concrete words (Barber et al., 2013). Thus, the concreteness N400 is believed to reflect embodied simulation process with sensory-motor recruitment. In addition to the concreteness N400 effect in metaphor processing, the P600/LPC component is also commonly found to index semantic reanalysis and integration process, with great significance to understand the specific association between P600/LPC and abstract semantics in verb metaphor comprehension. A few studies also found a distinct P600/LPC effect in the metaphor condition relative to the literal condition, suggesting that participants were aware of semantic conflicts 450–750 ms after encountering the unfamiliar metaphorical word and were able to integrate the abstract meaning of the action word into the sentence context *via* semantic reanalysis (Shen et al., 2015; Obert et al., 2018). In addition, these findings also suggest that both the sensorimotor system and the abstract sense processing system are involved in the verbal metaphorical processing, and the integration of metaphorical meaning is based on concrete simulation and abstraction. Based on the previous findings, the current study will further explore the activation time of concrete sense and abstract sense in verbal metaphor processing, which is related to N400 and P600/LPC, respectively.

In addition, previous studies have mainly focused on the timing of sensory-motion system activation in verbal metaphor comprehension (Lai et al., 2019), while the time course of metaphorical meaning integration has been underestimated. As verbal metaphors occur from the semantic conflict with literal meanings between verbs and context, the timing of the conflict is critical to the integration of metaphorical meanings. By manipulating animacy violation of the subject in the verbal metaphorical sentences, the previous study found that the metaphors with inanimate actor elicited an attenuated P600 as compared with the animate counterparts and converged to the same level as literal sentences (Ji et al., 2020). The result indicates that integration of metaphorical meaning is instantaneously accessed with the sentence meaning unfolding, thus the semantic conflict between verbs and different sentence components may lead to different time processes of metaphorical meaning extraction. Specifically, semantic conflict of verbal metaphors may occur between the verb and inanimate actors of the sentence, such as in subject-verb metaphor. It also appears when the object is presented, such as in verb-object metaphors. Therefore, it is to be examined whether the metaphorical meaning of subject-verb metaphors is integrated earlier than the verb-object metaphors. Since previous studies did not make a syntactic distinction between these two metaphors, it remains unclear that whether the semantic reanalysis and integration of abstract meaning are conducted once an action verb appears or it remains unfolded until the end of the sentence.

Taken together, we form the following two questions in the current study. First, in the processing of verbal metaphors, are the literal concrete meanings indicating sensorimotor simulations and/or the abstraction-related meanings activated? Second, at what time are these meanings integrated into the sentence? Answering these questions can further clarify the processing of verbal metaphors. Based on the above considerations, the current study aims to investigate the processing mechanism of verbal metaphors, focusing on the time course of metaphorical meaning integration. The N400 and P600/LPC components are used as indexes for the activation of associated sensorimotor simulation and abstract sense processing system. We assume that besides sensorimotor simulation, the comprehension of verbal metaphors also requires semantic abstraction. According to previous research, the N400 effect induced by verbs in verb-object metaphors will be similar to that in literal-concrete sentences, which recruits more perceptual motion simulation. And the P600/LPC effect elicited by verbs in subject-verb metaphors will be similar to that in literal-abstract sentences, reflecting the semantic aspects of processing, including processing and integration processes of abstract meaning.

Meanwhile, in the experiment, by setting the literal-metaphorical conflict, respectively, at the verb and the object, the timing of generating the metaphorical meaning is going to be distinguished. If the semantic integration of verbal metaphors occurs immediately with the unfolding of sentence meaning, then, participants will reanalyze the subject-verb metaphor sentences by integrating the meaning with the previous subject after the verb is presented, as well as access the appropriate metaphorical meaning of the sentence. In comparison, semantic reanalysis and integration will be triggered only when the object word of the verb-object metaphor is presented at the end of the sentence. Thus, we argue that the P600/LPC effect induced by the action verb tends to be revealed greater in the subject-verb metaphor sentences than that in the verb-object metaphor sentences.

## Materials and Methods

### Participants

*A priori* power analysis using G*Power 3.1 (Faul et al., 2007) suggested that, for a single-factor within-subject design with a power of 80%, approximately 24 participants for large effect size (d = 0.8) would be needed for our experiments. Additionally, we referred to the sample sizes used in previous studies (Lai et al., 2019; Ji et al., 2020), and consequently recruited 40 Chinese students as participants. All participants were completely unaware of the purpose of the present experiment. Written informed consent was obtained before the study. All participants had normal or corrected-to-normal vision, as established through self-report. The data for two participants were discarded from the statistical analysis as a result of excessive electroencephalogram (EEG) artifacts. Data of 38 healthy participants (17 male, 21 female; age 18–26 years; Mage = 20 years) were used for further analysis.

### Material

Some of the original materials were collected from the BCC (Beijing Language and Culture University-Corpus Center of China) and CCL (Peking University Modern Chinese Corpus) corpus, and the other part of the original materials are selected from the language of daily life and other genres of literature. The experiment contains four conditions of sentence materials (See Table 1): Subject-verb metaphorical sentences (SVM), Verb-object metaphorical sentences (VOM), literal-abstract sentences (LA) and literal-concrete sentences (LC). Subject-verb metaphor (SVM) condition referred to a sentence with an inanimate agent followed by an action verb that the agent could not physically perform. Therefore, the semantic conflict point falls on the verb of the sentence. In the verb-object metaphor (VOM) condition, the subject of the sentence is an animate agent, followed by an action verb and an abstract noun object. Since its object could not be physically manipulated, its conflict point falls on the object of the sentence. In the literal-abstract (LA) condition, the same inanimate agent was used, but the action verb was replaced by an abstract verb with a similar meaning. In the literal-concrete (LC) condition, the subject was an animate agent that could physically manipulate an object while the action verb remained the same as the verb in the metaphorical condition. Some of the sentences from the corpus were adapted for the consistency of SVO (NP1 + V + NP2) syntactic structure across all conditions, controlling the length of the sentence and visual complexity. And there were no statistical difference in words frequency of verb and object between conditions [verbs: (0.003 ± 0.003) vs. (0.004 ± 0.006); object: (0.011 ± 0.015) vs. (0.003 ± 0.006)].

**Table 1:** Sample stimuli.

Conditions	Examples	Explanation
Subject-verb metaphor (SVM)	剬司 / 抓住 / 机会。	The company grasped the opportunity.
Verb-object metaphor (VOM)	老板 / 抓住 / 机会。	The boss grasped the opportunity.
Literal-abstract (LA)	剬司 / 获得 / 机会。	The company sought the opportunity.
Literal-concrete (LC)	小哲 / 抓住 / 绳子。	Xiaozhe grasped the rope.

First, a questionnaire was adopted to evaluate and screen materials. The initial stimuli consisted of 80 sets of sentences. Two hundred and forty college students who would not participate in the formal experiment filled in the questionnaire to rate the acceptability, familiarity and comprehensibility of the experimental materials on a 5-point Likert scale (1 = completely unacceptable/highly unfamiliar/highly unintelligible; 5 = completely acceptable/highly familiar/highly intelligible). According to the evaluation results, 25 sets of sentences were selected for the formal experiment. The ratings of the four experimental sentences in acceptability, familiarity and comprehensibility were all above 3. In addition, 25 nonsense sentences were selected as filler materials, and the ratings of filler sentences in all dimensions were below 3.

We recruited 32 participants who neither took part in the evaluation experiment nor in the formal experiment to complete the sentence comprehension task and re-evaluate the experimental materials. The sentences were presented word by word on a computer screen. At the end of each sentence, the participants were asked to judge whether the sentence conveys a reasonable meaning, and press a corresponding key. One-way repeated-measures ANOVA of response time to the sentence comprehension task showed that there was no significant difference on RTs between the four experimental sentences *F* (3, 93) = 1.76, *p* = 0.16. The results suggested that the four experimental sentences were highly acceptable and understandable.

The experimental material consists of 5 lists, with each experimental condition from the same material set assigned to different material lists. Each list contains 25 sentences, of which five sentences per condition. Participants were randomly assigned to one of the lists.

### Procedure

**Figure 1:**
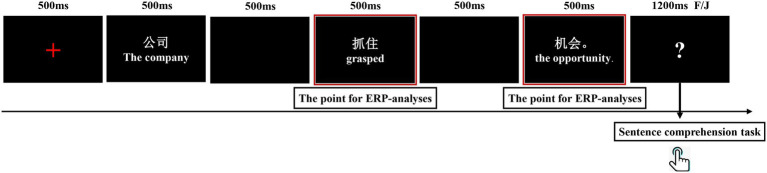
Trial structure of the experiment.

Participants were seated in a comfortable chair in a sound-attenuated, electrostatic-shielding room and instructed to read each sentence carefully. In the experiment, each sentence was presented word by word. First, participants performed a practice block of 10 sentences to get familiar with the task and experimental environment. The materials used in the practice stage would not appear in the formal experiment. Each trial began with a fixation point “+” displayed at the center of the screen for 500 ms, and then one sentence was displayed word by word in the center with white characters on a black background. Each word was displayed for 500 ms, with a 500 ms blank screen between words. The last word of the sentence was presented with a period. The participants were instructed to read each sentence silently and attentively. A sentence comprehension question appeared randomly after 40% of the trials during the experiment. The participants were asked to respond “meaningful” or “meaningless” as accurately and quickly as possible by pressing the “F” (meaningful) and “J” (meaningless) keys when “?” was presented, with “F” and “J” being balanced between subjects. If the participant did not press the key for 1,200 ms, the “?” automatically disappeared, and the next stimulus began. The whole experimental process lasted about 1.5 h. The trial structure is illustrated in Figure 1.

### EEG Recording and Analysis

We recorded EEG data from 64 electrodes (following the international 10–20 system) using a Neuroscan system and referenced to the left and right mastoids, with a ground electrode on the medial frontal aspect. The vertical electrooculogram (EOG) was recorded supra- and infraorbitally at the left eye; the horizontal EOG was recorded from the left versus the right orbital rim. The impedance of all electrodes was maintained at below 5 kΩ. The sampling rate was 1,000 Hz. Data were filtered online using a 0.05–100 Hz band-pass filter.

Offline processing of EEG signal data was performed in MATLAB using EEGLAB and ERPLAB toolbox. EEG data were re-referenced to the average of the left and right mastoids. A digital band-pass filter of 0.01–30 Hz was applied to the EEG recordings. Independent component analysis (ICA) was performed on continuous data for each participant to remove components relevant to eye movements and eye blinks. Epochs ranged from −200 to 800 ms after the onset of the stimulus, with the 200 ms interval preceding the stimulus onset serving as the baseline. Any epoch with EEG voltages exceeding a threshold of ±100 μV was excluded from the average.

In accordance with previous studies on N400 and P600 for metaphor (Shen et al., 2015; Lai et al., 2019) and the visual inspection based on the grand averaged data, we defined two time windows: 380–500 ms for the N400 and 670–770 ms for the P600 both for verb and object. Values were subjected to a 4 (sentence type: SVM, VOM, LA, LC) × 3 (electrode area: frontal, central, parietal) repeated-measures ANOVA. We classified these electrode sites into three areas: frontal (F3, Fz, F4), central (C3, Cz, C4), and parietal (P3, Pz, P4). All repeated-measures ANOVA results received Greenhouse–Geisser correction if the sphericity assumption was violated. Post-hoc multiple comparisons were carried out using Bonferroni-adjusted corrections. Effect sizes were presented as partial eta-squared ($\eta_{p}^{2}$) for *F* tests.

## Results

**Figure 2:**
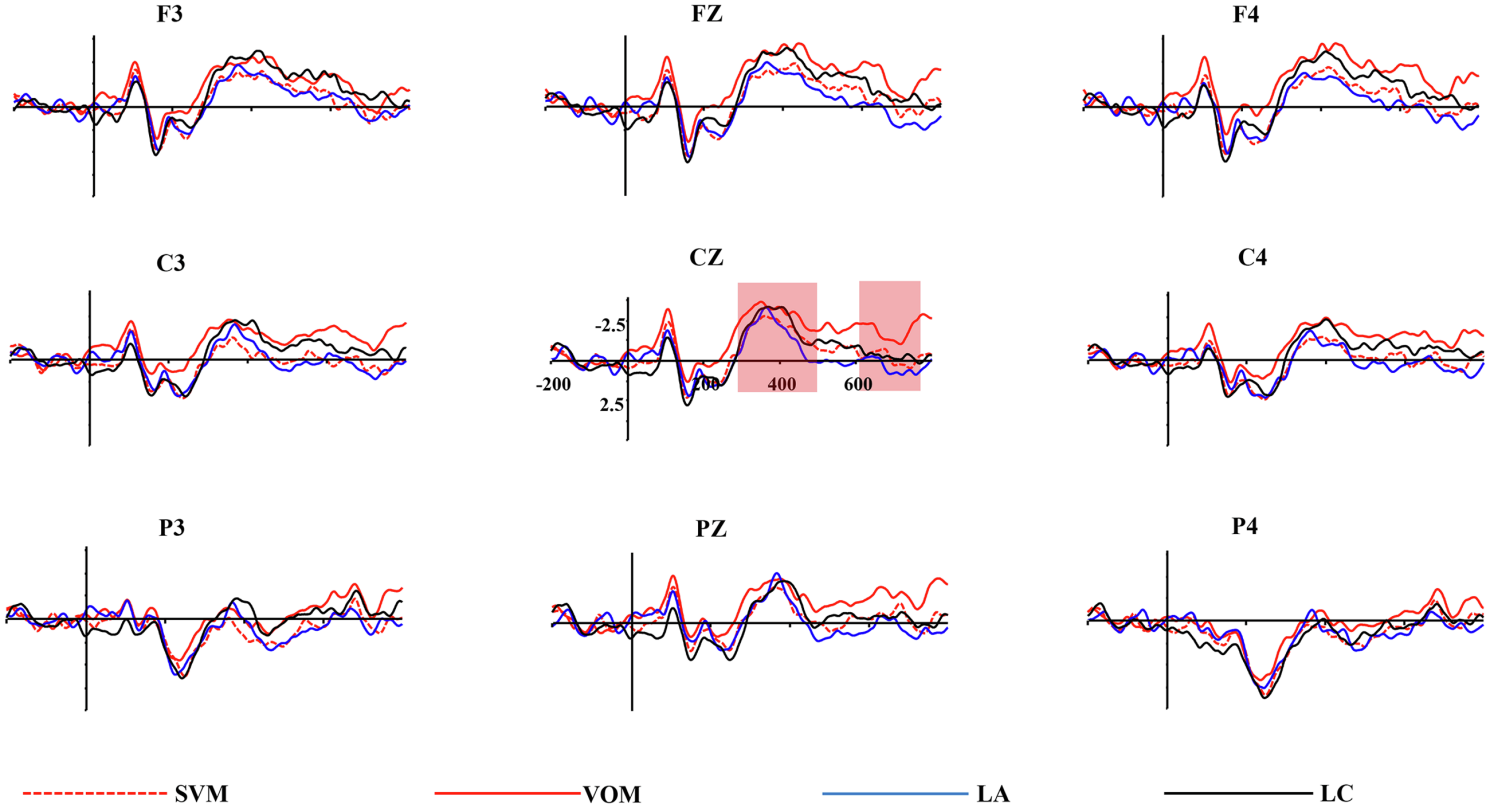
Grand average ERP waveforms recorded at verb for the chosen electrodes.

**Figure 3:**
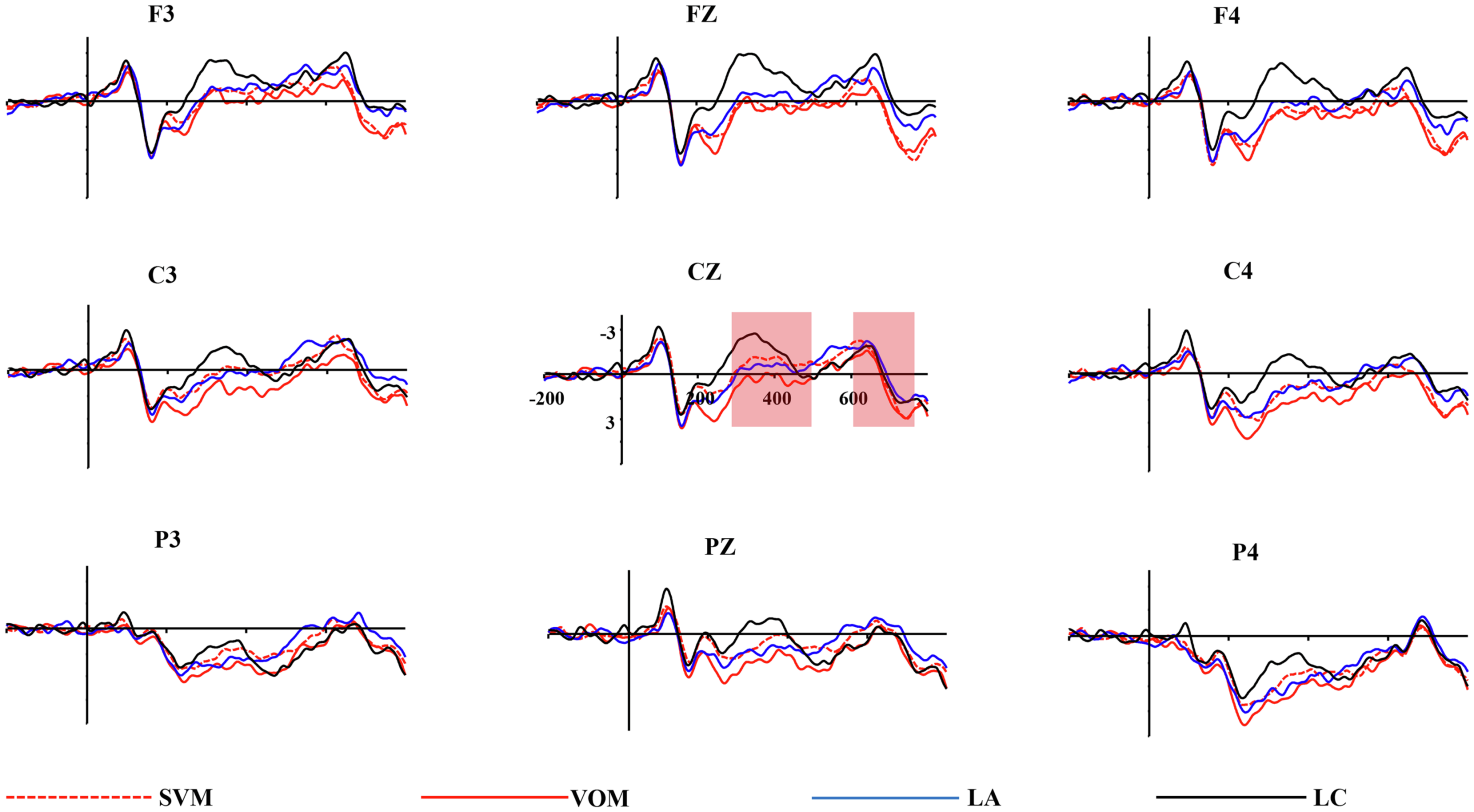
Grand average ERP waveforms recorded at object for the chosen electrodes.

The accuracy of the sentence comprehension questions was 88.5% (*SD* = 10.5%), indicating that the participants were engaged in reading. The total mean amplitudes for the target verb and object indicated that each condition induced significant N400 and P600/LPC (see Figures 2, 3).

### Verb Processing Stage

To investigate the processing mechanism and the activation time of concrete or abstract meaning of verbal metaphors, we analyzed the average amplitudes of N400 and P600/LPC detected when the verb appeared. The statistical results are shown in Table 2.

**Table 2:** Mean amplitude (*μV*) and standard deviation (*SD*) of N400 and P600/LPC for target verb.

EEG component	Electrode area	Sentence type			
		SVM	VOM	LA	LC
N400	Frontal	−1.70 ± 0.47	−2.83 ± 0.64	−1.46 ± 0.55	−2.47 ± 0.56
	Central	−0.90 ± 0.51	−2.00 ± 0.58	−0.67 ± 0.51	−1.63 ± 0.53
	Parietal	0.57 ± 0.45	−0.33 ± 0.46	0.55 ± 0.47	−0.09 ± 0.45
P600/LPC	Frontal	0.17 ± 0.34	−0.84 ± 0.60	0.66 ± 0.53	−0.47 ± 0.52
	Central	0.14 ± 0.34	−1.36 ± 0.53	0.57 ± 0.48	−0.39 ± 0.47
	Parietal	−0.15 ± 0.31	−1.22 ± 0.46	0.20 ± 0.44	−0.37 ± 0.45

#### N400

A repeated-measures ANOVA revealed significant main effects of both sentence type *F* (3, 111) = 3.38, *p* < 0.05, $\eta_{p}^{2}$ = 0.08, and electrode area *F* (2, 74) = 63.08, *p* < 0.001, $\eta_{p}^{2}$ = 0.63. Further analysis indicated that the waveforms for the verb-object metaphors and literal-concrete sentences were much more negative than those for the subject-verb metaphors and literal-abstract sentences, while there was no significant difference between verb-object metaphors and literal-concrete sentences. The mean amplitude of N400 decreased gradually in the frontal, central and parietal brain regions (*ps* < 0.05). No significant interaction was reported between sentence type and electrode.

#### P600/LPC

A repeated-measures ANOVA revealed a significant main effect of sentence type *F* (3, 111) = 3.86, *p* < 0.05, $\eta_{p}^{2}$ = 0.09, with the subject-verb metaphors and literal-abstract sentences inducing significantly more positive mean amplitudes than verb-object metaphors, while the mean amplitudes for subject-verb metaphors and literal-abstract conditions showed no significant difference. There was neither a main effect of electrode area nor the interaction between sentence type and electrode area.

### Object Processing Stage

To investigate the time course of semantic integration of metaphorical comprehension, we analyzed mean amplitudes of N400 and P600/LPC observed in the object processing stage when the sentence meaning was fully accessible. The statistical results are shown in Table 3.

**Table 3:** Mean amplitude (*μV*) and standard deviation (*SD*) of N400 and P600/LPC for target object.

EEG component	Electrode area	Sentence types			
		SVM	VOM	LA	LC
N400	Frontal	0.02 ± 0.62	0.35 ± 0.62	−0.35 ± 0.59	−1.05 ± 0.64
	Central	0.00 ± 0.56	1.03 ± 0.59	0.09 ± 0.56	−0.26 ± 0.58
	Parietal	1.44 ± 0.54	2.46 ± 0.59	1.72 ± 0.54	1.72 ± 0.51
P600/LPC	Frontal	1.75 ± 0.58	1.76 ± 0.53	0.45 ± 0.57	−0.08 ± 0.59
	Central	1.09 ± 0.52	1.31 ± 0.53	0.22 ± 0.48	0.39 ± 0.59
	Parietal	0.76 ± 0.48	0.93 ± 0.49	0.22 ± 0.42	0.80 ± 0.52

#### N400

A repeated-measures ANOVA revealed significant main effects of sentences type *F* (3, 111) = 3.42, *p* < 0.05, $\eta_{p}^{2}$ = 0.09, and electrode area *F* (2, 74) = 27.12, *p* < 0.001, $\eta_{p}^{2}$ = 0.42. Pairwise comparisons showed that the literal-concrete condition induced a stronger N400 effect than the other three conditions. The mean amplitude of N400 decreased gradually in the frontal, central and parietal brain regions. There was a significant interaction between sentence type and electrode area *F* (6, 222) = 3.27, *p* < 0.01, $\eta_{p}^{2}$ = 0.08. Simple-effect tests using *F*-test showed that the literal-concrete condition induced a larger N400 effect than the other three conditions in the frontal and central regions (*ps* < 0.05).

#### P600/LPC

A statistical analysis revealed a main effect of sentence type *F* (3, 111) = 3.08, *p* < 0.05, $\eta_{p}^{2}$ = 0.08. Pairwise comparisons showed that the waveform for the subject-verb metaphors was much more positive than that for the literal-abstract sentences, and the waveform for the verb-object metaphors was much more positive than that for the literal-concrete sentences, while there was no significant difference between the subject-verb metaphors and verb-object metaphors. There is an interaction between sentence type and electrode area *F* (6, 222) = 5.76, *p* < 0.001, $\eta_{p}^{2}$ = 0.14. Simple-effect tests using *F*-test showed that the subject-verb metaphors and verb-object metaphors elicited stronger P600/LPC effects than literal-concrete and literal-abstract conditions in the frontal region (*ps* < 0.05). There existed no main effect of electrode area.

## Discussion

The present study explored the neural mechanism and temporal processing of Chinese verbal metaphors from the sentence level. It was found that a similar N400 effect was elicited by the action verbs in the verb-object metaphorical and the literal-concrete sentences, both inducing more negative N400s than the subject-verb metaphors. Instead, the subject-verb metaphors elicited a more positive P600/LPC than the verb-object metaphors, which was similar to the literal-abstract sentences. When the object word was presented, both types of verbal metaphor induced a stronger P600/LPC effect than the literal sentences. Taken together, these results suggest that the verb-object metaphor activated more concrete meaning, while the subject-verb metaphor activated more literal-abstract meaning when the action word is processed. Moreover, in the final stage of semantic integration, both types of verbal metaphors need to be reanalyzed to ensure that the metaphorical meaning is successfully integrated into the whole sentence.

### Activation of Concrete and Abstract Meanings in Verbal Metaphors

The view of embodied semantics holds that verbal metaphors are strongly grounded in sensorimotor experience (Richardson et al., 2003; Gibbs, 2006; Wilson and Gibbs, 2007; Bardolph and Coulson, 2014). Accordingly, a verbal metaphor is processed as a simulation in which the activation of literal concrete meaning plays an important role. While other researchers argue that abstract semantic processing also has a crucial role in verbal metaphor comprehension, in addition to the activation of literal-concrete meaning (Jamrozik et al., 2016; Al-Azary and Katz, 2020). In line with the latter view, our findings found that the verb-object metaphors elicited an N400 effect similar to the literal-concrete condition, while the subject-verb metaphors induced a P600/LPC effect similar to the literal-abstract condition in the verb processing stage, confirming a combination of simulation and abstraction processing in verbal metaphor comprehension.

Recent studies confirm that the involvement of sensorimotor network and abstraction process is dynamic in metaphor comprehension (Al-Azary and Katz, 2020). Based on previous research, the present study further compares the activation of concrete and abstract meanings in the processing of subject-verb metaphors and verb-object metaphors. It shows that the N400 amplitudes induced by verbs in verb-object metaphorical sentences and literal-concrete sentences were greater than that in the subject-verb metaphors and literal-abstract sentences. Although both conditions contain the same action verb, there is no semantic conflict when processing the verb in a verb-object metaphor. Thus, the concrete meaning of the verb is activated in the first place. In contrast, a subject-verb metaphor produces such a conflict when the verb appears, in which the verb needed to be reanalyzed to activate a reasonable abstract meaning, namely, the metaphorical meaning of it. In the verb processing stage, the comprehension of a verb-object metaphorical sentence is more likely to deal with the literal-concrete meaning, relying on sensorimotor simulation, while processing a subject-verb metaphorical sentence activates the abstract meaning more quickly and extracts the metaphorical semantic relation between the subject and the verb.

In this study, by comparing the two types of verbal metaphors and the corresponding processing of literal-concrete and literal-abstract meanings, the results support the simulation-abstraction hybrid view, that is, both simulation and abstraction are viable mechanisms for processing metaphorical meaning (Al-Azary and Katz, 2020). Verbal metaphor comprehension is not entirely based on sensorimotor simulation but also counts on semantic abstraction. More important, the concrete and abstract semantic activation timeframes are regulated by the place where the semantic conflict lies.

### The Integration of the Metaphorical Meaning in Verbal Metaphors

The EEG activities in the verb processing phase demonstrated that both concrete and abstract meaning were activated in a verbal metaphor. However, the metaphorical meaning of the whole sentence remains uncertain at that stage, since the syntactic meaning has not been fully integrated yet. Especially for the verb-object metaphorical condition, only when the object appears can it constitute a semantic conflict to create a metaphorical meaning. Therefore, we further analyzed the ERP waveforms elicited by the object word in which the sentence meaning is being completely processed.

The results showed that in the object processing stage, both subject-verb metaphors and verb-object metaphors induced stronger P600 effect than literal sentences. When the object word is presented, the verb and object constitute a semantic conflict in both metaphorical conditions. Therefore, both types of metaphors need to be integrated with the context through the reanalysis of the sentence meaning, costing more cognitive resources than the processing of a literal sentence (Yang et al., 2013). In this phase, a verb-object metaphorical sentence creates a semantic conflict between the verb and the object, and the sentence meaning needs to be re-processed, resulting in a significant P600/LPC effect (Ji et al., 2020). While for a subject-verb metaphor, the abstract meaning of the verb has been activated during this period for the initial integration of metaphorical meaning, and semantic processing will continue with the unfolding of the sentence context until the end of the sentence. The pre-activation of the abstract sense of a subject-verb metaphor weakens the conflict between the verb and the object at the end of the sentence.

In addition, our findings are consistent with recent studies (e.g., Lai et al., 2019). The verb of subject-verb metaphorical sentence induced an N400 effect, and the very same sensory-motor recruitment in concrete literal language takes place for comprehending subject-verb metaphoric expressions. In other words, the specific action meaning of the verb at this stage is also activated, and only when the object finally appears, there shows a semantic conflict to be reintegrated with the previous context, so a P600/LPC effect similar to that induced by the verb-object metaphors is revealed. The P600/LPC effect also reflects that the metaphorical meaning of the sentence can be directly extracted and integrated through abstraction processing. By analyzing the semantic activation and integration at the different time windows of semantic processing, the study shows that the metaphorical meaning in the verbal metaphorical comprehension is instantly integrated and extracted as the meaning of the sentence is unfolded, and the time when semantic conflict appears will affect the activation time of concrete and abstract meanings in the metaphor.

Another interesting finding in this study is that the N400 elicited by literal-concrete condition in the frontal and central regions is larger than the other three conditions at the object processing stage. Possible reasons for this result are the noun’s imagery and/or concreteness effect (Weiland et al., 2014; Forgacs et al., 2015; Schmidt-Snoek et al., 2015). As N400 is associated with simulation and mental imaging of perceptual motion (Holcomb et al., 1999), semantic concreteness influences the N400 effect. Concrete nouns tend to elicit larger N400s than abstract nouns (West and Holcomb, 2000; Kanske and Kotz, 2007; Adorni and Proverbio, 2012; Barber et al., 2013). In the present study, only the object of the literal-concrete sentence is a concrete noun, which may lead to a stronger N400 effect in the frontal and central brain areas induced by the object of the literal-concrete sentence compared to the other three types of sentences.

## Conclusion

Based on the debate that whether the processing of verbal metaphor is relied entirely on perceptual motion simulation or involves abstract sense processing systems, the present study was designed to assess the cognitive mechanism of Chinese verbal metaphor processing, which is rarely investigated. Firstly, the processing mechanism of the metaphorical meaning of the verbal metaphor is a neural pattern that combines literal-concrete meaning and literal-abstract one, involving more cognitive resources than processing the above two meanings, respectively. It supports the simulation-abstraction hybrid view and thus has expanded the existing theoretical accounts of metaphor. Secondly, the processing of verbal metaphors is a dynamic process with gradual change. The concrete and abstract meanings are activated instantly according to the unfolding of sentence meaning at different stages, facilitating the extraction and integration of the metaphorical meaning of the expression. Therefore, the present findings can further clarify the processing process of the Chinese verb metaphor. Furthermore, the current research believes that when it comes to the mental representation of semantic embodiment, the processing of verbal metaphors should have cross-cultural consistency. The perceptual motion is also involved in the processing of Chinese verbal metaphors. However, when it comes to specific language characteristics, English language expressions are mostly abstract, while Chinese languages are characterized by image expressions. The differences of linguistic conventions may lead to the different performance of sensorimotor and abstract semantic involvement in the processing of Chinese verbal metaphors compared with English or other Indo-European languages characters. Specifically, although verbs in the verbal metaphorical sentences are concrete verbs, the activation of abstract characteristics is more dominant in English expressions (Tian et al., 2020), while in Chinese verbal metaphors, whether literal-concrete meaning will be processed prior has not been studied. The current study can enlighten whether verbal metaphors comprehension have cross-cultural consistency between different languages, and whether the perceptual motion system has a unique role different from that in the processing of Chinese verbal metaphors.

In addition, the findings of the present study on the neural processing mechanism of Chinese verbal metaphors in SOV sentences can provide some pedagogical implications for Chinese language teaching practices and learning. This study shows that the sensory-motor system is involved in the processing of Chinese metaphorical comprehension. Thus, the embodiment of language learning should be emphasized in Chinese teaching and learning. That is, language teachers and learners need to pay much attention to the use of multimodal resources, especially a variety of body movements, such as gesture and body posture, or embodied simulation of literal meanings. The use of these resources can enhance non-literal language learning and memory. In the process of Chinese metaphorical comprehension, besides the sensory-motion simulation of concrete concepts, the abstract sense stored in long-term memory will also be activated to promote the immediate integration of metaphorical meanings. Therefore, language learners should pay attention to the accumulation of knowledge in daily life to acquire figurative meanings more quickly.

However, there are limitations in the study. First, a limited amount of context is available before the target verb. Future studies using more extensive context (e.g., a complete sentence or multiple sentences) can potentially provide valuable insights into the effects of context and activation of meanings. Second, despite the high temporal resolution of event-related potentials (ERPs) technology used in the current study, its spatial resolution is not as accurate as other neuroimaging technologies, making it difficult to determine the specific brain regions activated during the comprehension of verbal metaphor. Therefore, subsequent studies need to further explore the neural mechanism of verbal metaphor processing with the help of other neuroimaging technology, to provide more direct evidence of brain activation for sensorimotor simulation. Third, this study did not distinguish or compare the embodied dimensions of verbs (e.g., verbs related to mouth, leg, hand, etc.), and the embodied dimensions involved were not comprehensive enough. Whether there would be differences in verb metaphors of different types of verbs could be further discussed in future studies. As such, future studies should consider syntax context, verb type and the variety of experimental methods.

## Data Availability Statement

The original contributions presented in the study are included in the article/Supplementary Material; further inquiries can be directed to the corresponding author.

## Ethics Statement

Ethical review and approval were not required for the study on human participants in accordance with the local legislation and institutional requirements. The patients/participants provided their written informed consent to participate in this study.

## Author Contributions

YL: conceptualization, methodology, investigation, formal analysis, writing - review & editing; XL: investigation, formal analysis, writing - original draft; YiW: writing - review & editing; HW: drafting the work and revising it critically for important intellectual content. YuW: conceptualization, funding acquisition, supervision, writing - review & editing. All authors contributed to the article and approved the submitted version.

## Funding

This work was supported by Project of Humanities and Social Sciences from Ministry of Education in China (20YJC190023) and Outstanding Young Scientific Research Team Project in humanities and social sciences of Zhengzhou University.

## Conflict of Interest

The authors declare that the research was conducted in the absence of any commercial or financial relationships that could be construed as a potential conflict of interest.

## Publisher’s Note

All claims expressed in this article are solely those of the authors and do not necessarily represent those of their affiliated organizations, or those of the publisher, the editors and the reviewers. Any product that may be evaluated in this article, or claim that may be made by its manufacturer, is not guaranteed or endorsed by the publisher.
