# Supplementary material for: Is the Processing of Chinese Verbal Metaphors Simulated or Abstracted? Evidence From an ERP Study
Source: Front Psychol. 2022 Jul 13;13:877997. doi: 10.3389/fpsyg.2022.877997 (PMC9328078; doi:10.3389/fpsyg.2022.877997)
Supplement: Supplementary file 1 [file Data_Sheet_1.pdf]

Appendix

25 sets of experimental materials

Conditions	Examples	Conditions	Examples
1. SVM	公司抓住机会。	8. SVM	春天播种希望。
1. VOM	老板抓住机会。	8. VOM	农民播种希望。
1. LA	公司获取机会。	8. LA	春天留下希望。
1. LC	小哲抓住绳子。	8. LC	农民播种庄稼。
2. SVM	行为打破规则。	9. SVM	祖国迈进光明。
2. VOM	选手打破规则。	9. VOM	患者迈进光明。
2. LA	行为违背规则。	9. LA	祖国获得光明。
2. LC	小红打破杯子。	9. LC	病人迈进病房。
3. SVM	酒吧点燃热情。	10. SVM	剧组丢掉信心。
3. VOM	小明点燃热情。	10. VOM	导演丢掉信心。
3. LA	酒吧激起热情。	10. LA	剧组丧失信心。
3. LC	小明点燃火柴。	10. LC	小雪丢掉垃圾。
4. SVM	学校花费时间。	11. SVM	军营打造队伍。
4. VOM	校长花费时间。	11. VOM	营长打造队伍。
4. LA	学校消耗时间。	11. LA	军营组建队伍。
4. LC	小莉花费金钱。	11. LC	军人打造兵器。
5. SVM	网络制造谣言。	12. SVM	政府加固信念。
5. VOM	同事制造谣言。	12. VOM	国民加固信念。
5. LA	网络散布谣言。	12. LA	政府巩固信念。
5. LC	厂家制造商品。	12. LC	士兵加固城墙。
6. SVM	商场推出广告。	13. SVM	杂志编织故事。
6. VOM	明星推出广告。	13. VOM	作者编织故事。
6. LA	商场发布广告。	13. LA	杂志撰写故事。
6. LC	爷爷推出车子。	13. LC	阿姨编织毛衣。
7. SVM	科学解开难题。	14. SVM	银行碰到麻烦。
7. VOM	教授解开难题。	14. VOM	行长碰到麻烦。
7. LA	科学破解难题。	14. LA	银行遭遇麻烦。
7. LC	小李解开扣子。	14. LC	小孩碰到桌子。

Conditions	Examples	Conditions	Examples
15. SVM	种族放下成见。	21. SVM	协会摘下头衔。
15. VOM	家长放下成见。	21. VOM	主席摘下头衔。
15. LA	种族舍弃成见。	21. LA	协会去掉头衔。
15. LC	小武放下书包。	21. LC	猴子摘下果子。
16. SVM	游戏糟蹋光阴。	22. SVM	集团交出权力。
16. VOM	小强糟蹋光阴。	22. VOM	股东交出权力。
16. LA	游戏浪费光阴。	22. LA	集团转让权力。
16. LC	小猛糟蹋食物。	22. LC	学员交出试卷。
17. SVM	社区争夺利益。	23. SVM	律所捂住秘密。
17. VOM	市民争夺利益。	23. VOM	律师捂住秘密。
17. LA	社区争取利益。	23. LA	律所隐藏秘密。
17. LC	孩子争夺玩具。	23. LC	小童捂住耳朵。
18. SVM	民族拾起传统。	24. SVM	市场嗅到危险。
18. VOM	国民拾起传统。	24. VOM	司机嗅到危险。
18. LA	民族继承传统。	24. LA	市场预知危险。
18. LC	老师拾起书本。	24. LC	厨师嗅到香气。
19. SVM	活动掀起风波。	25. SVM	法庭压住愤怒。
19. VOM	学生掀起风波。	25. VOM	妻子压住愤怒。
19. LA	活动引发风波。	25. LA	法庭平息愤怒。
19. LC	母亲掀起被子。	25. LC	小倩压住胳膊。
20. SVM	起义推翻制度。		
20. VOM	工人推翻制度。		
20. LA	起义反抗制度。		
20. LC	司机推翻车子。		
